# Supplementary material for: The induction of pyrenoid synthesis by hyperoxia and its implications for the natural diversity of photosynthetic responses in Chlamydomonas
Source: eLife. 2021 Dec 22;10:e67565. doi: 10.7554/eLife.67565 (PMC8694700; doi:10.7554/eLife.67565)
Supplement: Supplementary file 1. [file elife-67565-supp1.docx]

**SUPPLEMENTARY FILE 1**

**The Induction of a Pyrenoid by Hyperoxia: Implications for the Natural Diversity of Photosynthetic Responses in *Chlamydomonas***

Peter Neofotis^1^, Joshua Temple^1, 5^, Oliver L. Tessmer^1^, Jacob Bibik^1^, Nicole Norris^1^, Eric Poliner^1^, Ben Lucker^1^, Sarathi M. Weraduwage^1,3^, Alecia Withrow^2^, Barbara Sears^1^, Greg Mogos^1^, Melinda Frame^2^, David Hall^1^, Joseph Weissman^4^, and David M. Kramer*^1^

^1^MSU-DOE Plant Research Laboratory, Michigan State University, East Lansing, MI

^2^Center for Advanced Microscopy, Michigan State University, East Lansing, MI

^3^Great Lakes Bioenergy Research Center, Michigan State University, East Lansing, MI

^4^Corporate Strategic Research, ExxonMobil, Annandale, NJ

^5^Department of Plant Biology, Michigan State University, East Lansing, MI

*For correspondence: [kramerd8@msu.edu](mailto:kramerd8@msu.edu)

**Supplementary File 1A**: 2NBH Growth Media

| Volume of Stock Solution (ml/l) | Stock Solution | Stock Solution Content (grams) |
| --- | --- | --- |
| 2 | NaNO3 | 100 g/400 ml |
| 1 | CaCl_2_ | 10 g/ 400 ml |
| 1 | MgSO_4_ 7H_2_O | 30 g / 400 ml |
| 1 | TAP Phosphate Solution | 28.8 g K_2_HPO_4_ + 14.4g KH_2_PO_4_ /100ml |
| 1 | NaCl | 10 g/ 400 ml |
| 1 | Hutner Solution |  |

**Supplementary File 1B**: Rubisco activity rates (in picomole C fixed by rubisco s^-1^ per ug of Chl), showing effects of hyperoxia on activity of rubisco in CC-1009 and CC-2343. Raw extracts of the cells prior to (zero hours) and after exposure to hyperoxia (31 hours, see Materials and Methods) were assayed rapidly (hatched bars), reflecting the native activation state, or after pre-incubation for 10 minutes in the presence of MgCl_2_, H^12^CO_3_^-^, and 6-phosphogluconate, which promotes reactivation of inhibited enzyme (solid bars) (See Figure 1 for graph). STD is the standard deviation of the three biological replicates, each with three technical replicates.

**Supplementary File 1C**: Rates of oxygen evolution in CC-2343 and CC-1009 (μM O_2_ min^-1^) and maximum oxygen compensation point in control and cells pre-treated for 6 hours with hydrogen peroxide (see Figure 14 for graphs).
