# Supplementary material for: The induction of pyrenoid synthesis by hyperoxia and its implications for the natural diversity of photosynthetic responses in Chlamydomonas
Source: eLife. 2021 Dec 22;10:e67565. doi: 10.7554/eLife.67565 (PMC8694700; doi:10.7554/eLife.67565)
Supplement: Transparent reporting form [file elife-67565-transrepform1.docx]

***eLife’s* transparent reporting form**

We encourage authors to provide detailed information *within their submission* to facilitate the interpretation and replication of experiments. Authors can upload supporting documentation to indicate the use of appropriate reporting guidelines for health-related research (see [EQUATOR Network](http://www.equator-network.org/%20)), life science research (see the [BioSharing Information Resource](https://biosharing.org/)), or the [ARRIVE guidelines](http://www.plosbiology.org/article/info:doi/10.1371/journal.pbio.1000412) for reporting work involving animal research. Where applicable, authors should refer to any relevant reporting standards documents in this form.

If you have any questions, please consult our Journal Policies and/or contact us: [editorial@elifesciences.org](mailto:editorial@elifesciences.org).

**Sample-size estimation**

- You should state whether an appropriate sample size was computed when the study was being designed
- You should state the statistical method of sample size computation and any required assumptions
- If no explicit power analysis was used, you should describe how you decided what sample (replicate) size (number) to use

Please outline where this information can be found within the submission (e.g., sections or figure legends), or explain why this information doesn’t apply to your submission:

An appropriate sample size was used in our experiments. This questions seemed geared toward epidemiological or health studies, and our answers for specific figures are closely related in the next query about replicates. We shall answer in detail about our main figures there.

**Replicates**

- You should report how often each experiment was performed
- You should include a definition of biological versus technical replication
- The data obtained should be provided and sufficient information should be provided to indicate the number of independent biological and/or technical replicates
- If you encountered any outliers, you should describe how these were handled
- Criteria for exclusion/inclusion of data should be clearly stated
- High-throughput sequence data should be uploaded before submission, with a private link for reviewers provided (these are available from both GEO and ArrayExpress)

Please outline where this information can be found within the submission (e.g., sections or figure legends), or explain why this information doesn’t apply to your submission:

In our examination of the effects of hyperoxia on the pyrenoid starch sheath, for each treatment, samples were taken from three different bioreactors (in actuality, we sampled even more than this, because we had preliminary tests that were consistent, and subsequent consistent runs for other tests where we also examined cells). During the treatment, dozens of cells were viewed via light microscopy, confirming our hypothesis that hyperoxia induced pyrenoid formation. For the TEM, dozens of cells were examined, and at least two dozen of each strain for each treatment photographed. Representative images were chosen for the paper, and the supplementary material also contains many images. But, for transparency, we are also including additional exemplary images in a power point showing the effects of hyperoxia at 6 hours (See Transparent Reporting Figures 1, 2, 3, 4, 5, 6 , 12).

The cumulative biomass measurements (Figure 3) represent, as stated in the figure caption, the average of three different biological replicates (each biological replicate being a different bioreactor and so culture of algae). The growth assay on TAP plates (Appendix 1-Figure 7) show clearly the three replicate tests on each plate.

Because the experiment demonstrating that H_2_O_2_ induced the pyrenoid was something that we felt would be of much interest, and because we are preparing for future examinations with the same assay, the experiment has now been done at least 6 times with each strain, CC1009 and CC2343, with replicates run each time. The results are very consistent and clear even with light microscopy. The morphometric analysis was reported in Figure 5. The figure caption details how many cells were analyzed. (See Transparent Reporting Figure 7, 8, 9, 10 for additional images).

The figure caption in Figure 6 details how many cells were examined for each treatment to look for the localization of rubisco. This experiment was repeated on a different culture and day, and examined with a different confocal microscope, with similar results (Appendix 1-Figure 17). We also repeated the experiment with the original microscope (Transparent Reporting Figure 11).

The cells photographed in Figure 7 are exemplary images from experiments repeated three separate times, each with very similar results.

The data from Figure 8 represents assays done on three different cultures exposed to hyperoxia, each one constituting a biological replicate.

The images in Figure 9 are exemplary images from three biological replicates for each treatment.

The data in Figure 10 also represents three biological reps for each strain.

**Statistical reporting**

- Statistical analysis methods should be described and justified
- Raw data should be presented in figures whenever informative to do so (typically when N per group is less than 10)
- For each experiment, you should identify the statistical tests used, exact values of N, definitions of center, methods of multiple test correction, and dispersion and precision measures (e.g., mean, median, SD, SEM, confidence intervals; and, for the major substantive results, a measure of effect size (e.g., Pearson's r, Cohen's d)
- Report exact p-values wherever possible alongside the summary statistics and 95% confidence intervals. These should be reported for all key questions and not only when the p-value is less than 0.05.

Please outline where this information can be found within the submission (e.g., sections or figure legends), or explain why this information doesn’t apply to your submission:

We used standard statistic hypothesis testing. Figure 1 details that “error bars present the standard deviation of three biological replicates, each with three technical replicates.” For Figure 3, the error bars represent standard deviation for three separate reactor experiments, as noted in the figure caption. Similarly, for Figure 8, the error bars represent the standard deviation among three biological replicates, as noted in the figure caption. For Figure 10, the shading represents the 95% confidence interval of the three replicates, as noted in the figure caption. All the graphs in the figure captions in Appendix 1 similarly contain specifics about the statistics used. The Methods (under the Microscopy subheading) detail the methods used in our morphometric analysis.

(For large datasets, or papers with a very large number of statistical tests, you may upload a single table file with tests, Ns, etc., with reference to sections in the manuscript.)

**Group allocation**

- Indicate how samples were allocated into experimental groups (in the case of clinical studies, please specify allocation to treatment method); if randomization was used, please also state if restricted randomization was applied
- Indicate if masking was used during group allocation, data collection and/or data analysis

Please outline where this information can be found within the submission (e.g., sections or figure legends), or explain why this information doesn’t apply to your submission:

For functional or growth experiments, groups were systematically allocated by algal strain and treatment, randomly selecting the bioreactors for each strain and condition. Imaging of TEM sections for morphometric or quantification was performed by co-author, AW. To eliminate bias, AW was not made aware of the culture conditions or the expected phenotypes for each sample. To gather a wide range of representative samples, micrographs were recorded for a series of fields of view across multiple samples. For the micrographs, the positioning of the microscope was situated with as little bias as possible, and any and all cells in the in-focus field of view were recorded and subsequently measured. Only cells that were clearly damaged by fixation or sectioning were eliminated. This procedure was repeated with multiple fields of view across multiple grids.

**Additional data files (“source data”)**

- We encourage you to upload relevant additional data files, such as numerical data that are represented as a graph in a figure, or as a summary table
- Where provided, these should be in the most useful format, and they can be uploaded as “Source data” files linked to a main figure or table
- Include model definition files including the full list of parameters used
- Include code used for data analysis (e.g., R, MatLab)
- Avoid stating that data files are “available upon request”

Please indicate the figures or tables for which source data files have been provided:

All the raw figure files, the data for our graphs, and the python notebooks to make our graphs are available on a github site. We are also providing many additional TEM images in the Voluntary Reporting Files, and those raw images are also available on the github site.

<https://github.com/protonzilla/Neofotis2021_Pyrenoid_Hyperoxia>

On the site, we also have a power point

H2O2_Pyrenoid_Paper_MicroscopyImagesTagged_v7.pptx

with all the images tagged with their file names. We also include additional images (Transparent Reporting Images) at the end of the power point. All these raw images are also available on the github site, in the appropriate folder.
